# Predicting adherence to COVID-19 preventive measures among South Korean adults aged 40 to 69 Years using the expanded health empowerment model

**DOI:** 10.1016/j.ssmph.2023.101411

**Published:** 2023-04-24

**Authors:** Su-Jung Nam, Tae-Young Pak

**Affiliations:** Department of Consumer Science and Convergence Program for Social Innovation, Sungkyunkwan University, Seoul, South Korea

**Keywords:** Health empowerment, Knowledge–attitude–behavior model, Health belief model, Preventive behavior, COVID-19

## Abstract

The public health environment in South Korea is advancing toward the late stages of the COVID-19 pandemic. However, there is limited knowledge about the extent of individuals' compliance with preventive measures during this transitional period and the potential predictors that determine such compliance behaviors. In this study, we employed the expanded health empowerment model to investigate factors associated with COVID-19 preventive behaviors among Korean adults in late 2022. Our theoretical framework integrates the knowledge-attitude-behavior model with the health belief model to conceptualize health empowerment underlying the formation of preventive behaviors. We collected data from 1100 Korean adults aged 40–69 years through an online survey conducted in October 2022. Participants responded to questions about their knowledge of COVID-19, attitudes towards the disease, adherence to preventive measures, infection history, and sociodemographic characteristics. Structural equation modeling was employed to assess the relationships between knowledge, attitudes, and preventive behaviors related to COVID-19. Results showed that attitudes toward the disease predict adherence to preventive behaviors. We also found that COVID-19 knowledge partially determined the attitudes toward the disease. However, COVID-19 knowledge was not directly associated with adherence to preventive behaviors. Additionally, the associations between knowledge, attitudes, and preventive behaviors did not differ between infected and never-infected individuals. Overall, this study finds empirical support for the expanded health empowerment model, which connects knowledge to preventive behaviors through positive attitudes toward the disease, while underscoring the limited role of infection history in this association. These findings can help policymakers understand individual responses to public health guidelines in the late pandemic era and develop policies to mitigate further transmission of COVID-19.

## Highlights

• Examined factors influencing COVID-19 preventive behaviors in late pandemic era.
• Proposed the expanded health empowerment model to conceptualize preventive behaviors.
• Knowledge of COVID-19 affects preventive behaviors via attitudes toward disease.
• The knowledge-attitudes-behavior link does not differ by infection history.

## 1 Introduction

The third year of the pandemic began with medical innovations, such as vaccines and antiviral drugs, to counter the transmission of the novel coronavirus (COVID-19). The emergence of highly infectious but less severe variants, like *Delta* and *Omicron*, contributed to the view that COVID-19 would be gradually contained and subdued (Adam, 2022). Despite occasional flare-ups in confirmed cases across regions (Phillips, 2021), people no longer seem to be excessively worried about contact with the coronavirus and the related mortality consequences (Jackson, Newall, Duran, & Golden, 2022). As of April 2022, an estimated 80%–85% of people in South Korea had received a booster vaccine or acquired disease-induced immunity against COVID-19 (Jang et al., 2022). The daily rate of confirmed cases peaked around March 2022 and has rapidly declined since then (Lim & Sohn, 2022).

Researchers and policymakers have begun discussing the transition from the pandemic to the endemic phase of COVID-19 (Gostin, 2022). Endemic is an epidemiological term indicating “the constant presence or usual prevalence of an infectious agent in a population within a geographic area” (Center for Disease Control and Prevention, 2012). It is different from a pandemic, as the disease is limited to a particular region and can be controlled by the existing healthcare infrastructure (Phillips, 2021). Several developed countries are working on updated management procedures, labeled as “living with COVID-19″ policies (Nature, 2022). The transition toward the endemic stage may require people to learn how to live with the coronavirus and adopt appropriate mitigation strategies to reduce the risk of infection. Public health guidelines will likely resemble prevention strategies for seasonal influenza, which recommend regular vaccination and daily practices to minimize virus exposure (Center for Disease Control and Prevention, 2021).

Throughout the pandemic, compliance with suggested guidelines has been central to slowing the spread of COVID-19 (West, Michie, Rubin, & Amlôt, 2020). In early 2020, the World Health Organization announced several prevention strategies, including (a) washing hands, (b) wearing a mask, (c) physical distancing, (d) self-isolation and quarantine, (e) not touching the face, and (f) avoiding large group gatherings (World Health Organization, 2020). Prior literature has shown that individual adoption of preventive measures varies with one's understanding of the virus and the ability to change behaviors accordingly (Jang & Hwang, 2022; Nam, 2023). For instance, Wise, Zbozinek, Michelini, Hagan, and Mobbs (2020) found that the perceived probability of being infected predicts engagement in physical distancing and handwashing. Jang (2022) revealed that knowledge of the disease and acquaintance with related government policies lead to adequate compliance with preventive health guidelines. Several studies have used the health belief model (HBM) to show that people's beliefs about COVID-19, as measured by perceived susceptibility, perceived severity, perceived benefit, and perceived barriers, predict adherence to preventive measures (Alagili & Bamashmous, 2021; Badr et al., 2021; Butty et al., 2022; Guidry et al., 2021; Jose et al., 2021; Karimy et al., 2021; Mirzaei et al., 2021; Moghadam, Zobeidi, Sieber, & Löhr, 2022; Noghabi, Mohammadzadeh, Yoshany, & Javanbakht, 2021; Tong, Chen, Yu, & Wu, 2020; Yan, Lai, Lee, & Ng, 2021).

The COVID-19 situation in South Korea has changed dramatically since the emergence of the *Delta* and *Omicron* variants (Jeon, Han, Kim, & Lee, 2022). Despite the surge in confirmed cases, the Korean government has lifted stringent containment measures, such as public lockdowns and business hour restrictions, and implemented the exit strategy for the pandemic (Lim & Sohn, 2022). While individuals were advised to follow public health guidelines on personal hygiene and preventive practices, governmental efforts have focused on reducing the mortality risk for high-risk groups, including older adults and disadvantaged families (Lim & Sohn, 2022). This paradigm shift in mid-2022, called the “returning to normalcy” plan (Nham et al., 2022), may have altered the perceived risk of COVID-19 and, consequently, individual behaviors to reduce infection risk (Bíró, Branyiczki, & Elek, 2022). Whether the public's adherence to preventive measures has changed since the easing of restrictions has yet to be fully understood.

The transition from the pandemic to the endemic phase does not imply that COVID-19 is no longer a threat to public health. COVID-19 mitigation strategies may be redesigned with some relaxation, but their effectiveness still depends on the public to curb occasional seasonal flare-ups (Gostin, 2022). The concept of health empowerment suggests that engagement in health-promoting behaviors is shaped by knowledge, beliefs, and attitudes toward diseases (Bettinghaus, 1986). As the pandemic continues, people accumulate knowledge about the health consequences of COVID-19 infection and experience changes in their attitudes and beliefs toward prevention strategies. Despite the expected behavioral changes during the transition, little is known about the psychological processes leading to preventive health behaviors in the late pandemic era. To fill this research gap, the current study examines the associations between knowledge of COVID-19, attitudes, and beliefs, and adoption of mitigation strategies using data on Korean adults collected in late 2022. The knowledge–attitude–behavior (KAB) model is expanded with the HBM to conceptualize health empowerment underlying knowledge acquisition, belief generation, and preventive behavior among Korean adults. The results of this study contribute to the growing literature on individual compliance with COVID-19 mitigation measures and offer implications for designing prevention strategies in the endemic era.

## 2 Conceptual framework

Empowerment is the process of becoming competent and proactive in controlling one's life (Rappaport, 1987). Early conceptualizations of empowerment focused on the ability to take control of an outcome of interest (Rappaport, 1987) and identified knowledge and skills as the necessary components of empowerment (McClelland, 1973). Dubois (1993) suggested that for people to be empowered, they need to gain knowledge of themselves and their surrounding environments, as well as develop skills and attitudes to successfully perform tasks in various situations. In public health, empowerment is defined as “a process through which people gain greater control over decisions and actions affecting their health” (World Health Organization, 1998, p. 6). Empowerment in the health domain requires empowering patients with the knowledge, attitudes, and behaviors needed to achieve the desired level of health (Nam, 2021). The Ottawa Charter for Health Promotion notes that health empowerment is an integral part of “taking control of things that determine health” and “achieving the fullest health potential” (World Health Organization, 1986, p. 1). Nam (2023) showed that digital empowerment of older adults significantly affects their COVID-related knowledge and behavior scores. Moreover, Mark, Udod, Skinner, and Jones (2022) highlighted the need for enhanced educational resources aimed at underprivileged populations and communities, especially among less-educated groups that display lower levels of COVID-19 knowledge. The KAB model, proposed by Bettinghaus (1986), is a systematic investigation of health empowerment.

**Fig. 1:**
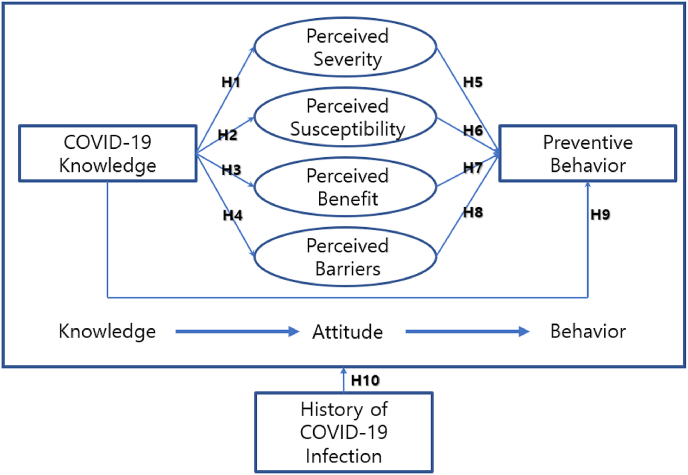
Conceptual model and hypotheses.

The KAB model theorizes that health-related knowledge influences an individual's attitudes and beliefs, which subsequently impacts their health behavior. This model is closely linked to learning theory (Bandura, 1976) and the diffusion of innovation theory (Roger, 1995), both of which assert that knowledge must be paired with a volitional element to promote behavioral change. Studies on vaccination acceptance (Pan, He, Lin, & Zhong, 2022; Zheng, Jiang, & Wu, 2022) and the adoption of prevention strategies (Deng et al., 2006; Jalloh et al., 2017) have provided empirical support for the KAB model. Additionally, the model serves as a conceptual foundation for understanding how health education and promotion lead to positive health behaviors and outcomes (Kemm & Close, 1995). In the KAB model, knowledge and behavior are evaluated as objective indicators, while attitudes are assessed through various methods (Lai et al., 2021; Miller, Booraem, Flowers, & Iversen, 1990; Morrison, Baker, & Gillmore, 1994). Previous studies have emphasized the significance of beliefs in measuring attitudes (Ajzen, 1993; Eagly & Chaiken, 1993; Erwin, 2001). Zhang, Chen, and Zhang (2022) proposed an expanded KAB model in which health literacy affects COVID-19 health behaviors through the four domains of health beliefs from the HBM. Reinhardt, Weber, and Rossmann (2022) also used HBM to measure attitudes when applying a theory of planned behavior to voluntary compliance with COVID-19 preventive measures. The present study proposes a similar mediation model, as depicted in Fig. 1.

The HBM was developed to understand why people do or do not adopt disease preventive strategies or medical screening tests (Hochbaum, 1958). It originated from the expectancy–value theory, which states that threat perception and the expected threat reduction potential of a recommended action lead to changes in health behaviors (Rosenstock, 1974). The HBM states that people engage in health-promoting behaviors when they feel that they have a high chance of acquiring a disease (perceived susceptibility) and/or expect severe medical and social consequences (perceived severity). It further assumes that one's engagement intention is influenced by the efficacy of health behaviors in reducing disease risks (perceived benefit) and the perceived obstacles to behavioral changes (perceived barriers) (Rosenstock, 1974). The model was later expanded with two additional constructs to reflect the observations that health behaviors could be altered by external and internal cues that motivate one to adopt certain behaviors (cues to action) and the confidence in one's ability to successfully perform a behavior (self-efficacy) (Rosenstock, Strecher, & Becker, 1988). The HBM has been shown to predict compliance with recommended practices to prevent COVID-19 infection during the early stages of the pandemic (Alagili & Bamashmous, 2021; Badr et al., 2021; Butty et al., 2022; Guidry et al., 2021; Jose et al., 2021; Karimy et al., 2021; Mirzaei et al., 2021; Moghadam et al., 2022; Noghabi et al., 2021; Tong et al., 2020; Yan et al., 2021).

Replacing attitude in the KAB model with the four constructs of the HBM, the current study proposes an expanded health empowerment model for predicting COVID-19 preventive behaviors (Fig. 1). This model identifies COVID-19 knowledge as a crucial aspect of health beliefs and assumes directional paths leading to perceived severity (H1), perceived susceptibility (H2), perceived benefit (H3), and perceived barriers (H4). The four HBM components and the underlying knowledge are further hypothesized to predict compliance with COVID-19 preventive measures, resulting in hypotheses H5–H9. Additionally, a multi-group analysis was conducted to examine differences in the associations between knowledge, attitudes, and preventive behaviors between infected and never-infected individuals (H10). The effect of infection history is theoretically ambiguous because recovery from COVID-19 might have granted immunity and reduced motivation for preventive behavior (Malesza & Kaczmarek, 2021), or the threat of losing immunity and becoming re-infected could motivate individuals to maintain compliance with preventive measures (Ghorbani et al., 2022). To date, epidemiological data on individuals who have recovered from COVID-19 have been unavailable for analyzing preventive health behaviors. This study addresses this gap in the literature by considering both infected and never-infected individuals and explicitly modeling the differences in COVID-19 preventive behaviors by infection history.

## 3 Methods

### 3.1 Data

This study analyzed data collected by Macromill Embrain (https://embrain.com), an independent market research company based in Seoul, South Korea. The survey was conducted online in October 2022. Participants were recruited from Embrain's panel database of over a million individuals. Our study sample was restricted to individuals aged 40 to 69, a sample that excludes retirees and younger adults who are not eligible for preventive medical services provided by the national health insurance. This selection criterion omits older and younger adults whose health behaviors may differ from middle-aged adults in unobserved ways and enables us to identify a more homogeneous sample in terms of health characteristics. A total of 1100 participants were randomly selected based on their demographic characteristics (age, gender, and region), and written informed consent was obtained before the survey. Participants who fully completed the survey were compensated at a rate of 100 KRW (approximately 0.1 USD) per minute.

### 3.2 Participant characteristics

Table 1 presents the descriptive statistics of the study sample. Among the 1100 participants aged 40–69 years, approximately 50.9% were female, 49.1% were male, and 47.3% were full-time workers. The majority of respondents had a high school diploma or college degree (98.6%) and were in a marital relationship (96.5%). Monthly income (in 10,000 KRW) was predominantly in the 201–300 (20.1%) and 301–400 categories (18.5%), with subjective economic status mainly being middle-low (48.0%) and middle-high (30.7%). About 50.5% of the participants rated their health as average, while 32.7% reported being in good health. Lastly, the proportion of participants with a history of COVID-19 infection was 38.7%.

**Table 1:** Sample characteristics.

Category	Frequency	%
Gender		
Male	540	49.1
Female	560	50.9
Age		
40-49	380	34.5
50-59	360	32.7
60-69	360	32.7
Education		
Elementary school graduated	4	0.4
Middle school graduate	11	1.0
High school graduate	284	25.8
College graduate	693	63.0
Post graduate	108	9.8
Work type		
Self-employed	105	9.5
Full-time worker	520	47.3
Part-time worker	105	9.5
Housewife	219	19.9
Inoccupation	40	3.6
Retirement	88	8.0
Others	23	2.1
Marital status		
Yes	937	96.5
No	163	3.5
Monthly income (in 10000 KRW)^a^		
≤ 100	100	9.1
101-200	149	13.5
201-300	221	20.1
301-400	204	18.5
401-500	148	13.5
501-600	106	9.6
601-700	63	5.7
700 ≤	109	9.9
Subjective Economic status		
Low-low	51	4.6
Low-high	161	14.6
Middle-low	528	48.0
Middle-high	338	30.7
High-low	20	1.8
High-high	2	0.2
Self-rated health		
Very poor	12	1.1
Poor	163	14.8
Average	555	50.5
Good	360	32.7
Very good	10	0.9
History of COVID-19 infection		
Yes	426	38.7
No	674	61.3

Notes.

a 1 USD = 1309.5 KRW (as of March 20, 2023).

### 3.3 Measure

The structured questionnaires were developed based on the relevant literature (Janz & Becker, 1984; Kim, Kim, Yang, & Chae, 2021; Korea Disease Control and Prevention Agency, 2022; Rimal & Real, 2003) (Table 2). Knowledge was calculated as the sum of correct responses minus the sum of incorrect answers, following the methodology of Reinhardt et al. (2022). Participants answered six true-or-false statements about COVID-19-related facts obtained from the Korea Disease Control and Prevention Agency (2022). They indicated whether each statement was true, false, or if they were unsure (−6 = "all answers wrong,” 6 = "all answers correct").

**Table 2:** Measurement items.

	Correct response
**Knowledge**	
K1. There is no effective treatment for COVID-19, but treatment to relieve symptoms may help most patients recover	Truth
K2. COVID-19 only leads to severe disease in older adults, people with chronic diseases, or people with obesity	False
K3. Children and adolescents do not need to take COVID-19 precautions	False
K4. COVID-19 can also be transmitted through mosquitoes	False
K5. COVID-19 is spread through droplets from an infected person	Truth
K6. COVID-19 is related to temperature change	False
	Mean (*SD*)
**Attitude**	
***Perceived severity***	3.37 (.71)
A1. COVID-19 is fatal disease	
A2. COVID-19 is painful disease	
A3. I would be disappointed and shocked if I get COVID-19	
A4. I think the time and economic loss caused by COVID-19 are huge	
***Perceived susceptibility***	4.01(.53)
A5. Anyone can get COVID-19	
A6. My family can get COVID-19	
A7. People around me, including my friends and colleagues, can get COVID-19	
***Perceive benefit***	3.12 (.42)
A8. Prevention should be a top priority to avoid getting infected with COVID-19	
A9. I think preventive behaviors against COVID-19 are effective	
A10. Preventing COVID-19 is necessary	
A11. Preventing COVID-19 is important	
***Perceived barriers***	2.89 (.81)
A12. It is cumbersome and annoying to have to go to the hospital to take preventive actions against COVID-19	
A13. The cost of preventive behavior against COVID-19 is burdensome	
A14. I am afraid that I will be diagnosed as a patient after being tested for COVID-19	
**Behavior**	3.98 (.54)
B1. I wear a well-fitting mask	
B2. I do not go to crowded places or places with poor ventilation.	
B3. I wash my hands often	
B4. I monitor my health every day	

Attitude was assessed using the four constructs of the HBM (perceived severity, perceived susceptibility, perceived benefit, and perceived barriers) on a 5-point Likert scale. The measurement items were sourced from previous studies (Janz & Becker, 1984; Rimal & Real, 2003) and adapted to the Korean context. The scale for perceived severity consists of four items: two capture the perceived severity of the disease, while the other two capture the emotional response to infection and its economic consequences. Perceived susceptibility was assessed using three items concerning the perceived likelihood of infection. Perceived benefit was measured using four items representing the perceived benefit of adhering to COVID-19 precautionary measures. Finally, the scale for perceived barriers include three items that prevent compliance with public health guidelines to prevent further infection. The mean score was obtained for the four subconstructs of the HBM, resulting in a 5-point score. The alpha coefficient of reliability for the four constructs ranged from 0.70 to 0.92.

Preventive behavior was measured using four items related to mask-wearing, avoiding large group gatherings, handwashing, and monitoring health conditions. These items were taken from the COVID-19 Prevention Scale developed by the Korea Disease Control and Prevention Agency in accordance with COVID-19 response guidelines (Kim et al., 2021). Participants evaluated their engagement in each preventive measure using a 5-point Likert scale. The measurement items had an alpha value of 0.74.

### 3.4 Analysis

Frequencies, percentages, and correlations were used to analyze the survey data on knowledge, attitudes, and behaviors related to COVID-19. The validity and reliability of the constructs were evaluated using confirmatory factor analysis. This study utilized factor loadings, construct reliability, average variance extracted (AVE), and the square root of AVE to confirm the suitability of the constructs. After validating the measurement model, a structural equation model (SEM) was applied to assess the path relationships among the constructs. The SEM is a multivariate modeling technique used to estimate the complex relationships between a behavior in question and a set of constructs or factors in accordance with a conceptual framework. The estimates from the SEM were used to evaluate hypotheses H1–H10. Hypothesis testing was conducted at a significance level of 0.05. All statistical analyses were performed using SPSS 27.0 and AMOS 27.0.

## 4 Results

### 4.1 Measurement model assessment

The analysis begins with confirmatory factor analysis to validate the theory-driven attitudes measured using 14 measurement items. The model demonstrates a satisfactory fit, with χ^2^/df = 9.795, CFI = 0.902, and RMSEA = 0.090. Table 3 presents the assessment of the measurement model according to the construct reliability and convergent validity criteria (Hair, Hult, et al., 2019; Hair, Risher, Sarstedt, & Ringle, 2019). The constructs meet the required construct reliability, as their composite reliability values surpass the minimum threshold of 0.7, and all alpha values are greater than 0.6. Regarding convergent validity, this study examines the AVE. The results reveal that the AVE values range from 0.5 to 0.714 and thus exceed the acceptable level of 0.5. Consequently, we conclude that the four constructs possess convergent validity.

**Table 3:** Validity and reliability.

Construct	Indicator	Estimate	SE	t	p	AVE	CR	Cronbach's α
Perceived severity	A1	1.000				.501	.787	.782
	A2	.817	.046	17.704	***			
	A3	.996	.049	20.342	***			
	A4	.850	.044	19.277	***			
Perceived susceptibility	A5	1.000	1.000			.714	.907	.922
	A6	1.011	.018	56.589	***			
	A7	.806	.022	36.412	***			
Perceive benefit	A8	1.000				.586	.847	.848
	A9	.959	.030	32.505	***			
	A10	.606	.024	24.778	***			
	A11	.586	.028	20.823	***			
Perceived barriers	A12	1.000				.500	.742	.695
	A13	.785	0.59	13.257	***			
	A14	.527	.044	12.095	***			

Notes: SE, standard error; t, t-value; p, p-value; AVE, average variance extracted; CR, composite reliability. ***p < 0.01; **p < 0.05; *p < 0.1.

Table 4 presents the square roots of the AVE estimates on the diagonal and the correlations between the constructs below the diagonal. We find that all correlation coefficients are lower than the corresponding square roots of the AVE values, indicating acceptable discriminant validity according to the Fornell-Larcker criterion (Hair, Hult, et al., 2019; Hair et al., 2019).

**Table 4:** Discriminant validity.

	1.	2.	3.	4.
1. Perceived severity	**.708**			
2. Perceived susceptibility	.152	**.845**		
3. Perceive benefit	.202	.222	**.766**	
4. Perceived barriers	.149	.058	−.142	**.707**

Notes: Square roots of AVE estimates are on diagonal; correlations between constructs are below diagonal.

### 4.2 Structural model assessment

The results of the hypothesis verification are presented in Table 5. The structural model accounts for 18.9% of the variance in preventive behavior (adj. R^2^ = 0.189). The estimated structural model exhibits a good fit, with χ^2^/df = 14.672, CFI = 0.934, RMR = 0.094, and RMSEA = 0.015. Knowledge has a significant effect on perceived severity (β = −0.036, p < 0.01), perceived benefit (β = 0.023, p < 0.01), and perceived barriers (β = −0.054, p < 0.01). However, the effects of knowledge on perceived susceptibility and preventive behavior are not significant at the 5% level. Moreover, the effects of perceived severity (β = 0.072, p < 0.01), perceived susceptibility (β = 0.111, p < 0.01), perceived benefit (β = 0.417, p < 0.01), and perceived barriers (β = −0.113, p < 0.01) on behavior are all statistically significant.

**Table 5:** Estimation results of the structural model.

		Estimate	S.E.	*t*	*p*
H1	Knowledge → Perceived severity	−.036	.013	−2.761	***
H2	Knowledge → Perceived susceptibility	.009	.010	.955	.340
H3	Knowledge → Perceived benefit	.023	.008	3.027	***
H4	Knowledge → Perceived barriers	−.054	.015	−3.700	***
H5	Perceived severity → Behavior	.072	.022	3.350	***
H6	Perceived susceptibility → Behavior	.111	.029	3.889	***
H7	Perceived benefit → Behavior	.417	.037	11.367	***
H8	Perceived barriers → Behavior	−.113	.018	−6.157	***
H9	Knowledge → Behavior	−.007	.009	−.802	.422

Notes: S.E., standard error; t, t-value; p, p-value. ***p < 0.01; **p < 0.05; *p < 0.1.

### 4.3 Multi-group analysis based on COVID-19 infection history

Table 6 shows the results of the multi-group analysis comparing infected and never-infected individuals. The last column displays the p-values derived from testing the equality of path coefficients from two distinct SEM models. We determined that the differences in path coefficients based on infection history are not statistically significant at the 5% level. This indicates that the associations between knowledge, attitudes, and preventive behaviors were not influenced by the history of COVID-19 infection.

**Table 6:** Muti-group analysis based on COVID-19 infection history.

		Yes			No			Δχ^2^	p
		Estimates	*t*	*p*	Estimates	*t*	*p*		
H1	Knowledge → Perceived severity	−.031	−1.598	.110	−.037	−2.142	**	.041	.840
H2	Knowledge → Perceived susceptibility	−.014	−.954	.561	.021	1.703	*	3.356	*
H3	Knowledge → Perceived benefit	.011	.933	.351	.033	3.264	***	2.194	.139
H4	Knowledge → Perceived barriers	−.067	−3.079	***	−.0.45	−2.281	**	0.588	.443
H5	Perceived severity → Behavior	.042	1.170	.242	.086	3.172	***	.997	.318
H6	Perceived susceptibility → Behavior	.098	2.057	**	.118	3.146	***	0.098	.754
H7	Perceive benefit → Behavior	.370	5.947	***	.435	9.500	***	0.703	.402
H8	Perceived barriers → Behavior	−.161	−5.174	***	−.090	−3.964	***	3.327	*
H9	Knowledge → Behavior	−.016	−1.128	.256	−.003	−.236	.814	.526	.465

Notes: t, t-value; p, p-value. ***p < 0.01; **p < 0.05; *p < 0.1.

## 5 Discussion

The COVID-19 pandemic has prompted public health authorities to develop appropriate countermeasures to curb the spread of the disease. In the absence of a definitive treatment measure, individual compliance with preventive measures remains an important means of preventing and controlling COVID-19. In the present study, we examined the possible determinants of COVID-19 preventive behaviors among Korean adults during the late stages of the pandemic.

This study proposed the expanded health empowerment framework, which merges the KAB model with the HBM, to explain one's intention to engage in COVID-19 preventive measures. Our conceptual framework posits that knowledge of COVID-19 is the basis for establishing positive attitudes toward the disease and that such positive attitudes lead to compliance with suggested guidelines for preventing further infection with COVID-19. The four constructs that represented attitudes, namely, perceived severity, perceived susceptibility, perceived benefit, and perceived barriers, were significantly related to adherence to preventive behaviors (H5, H6, H7, and H8 were supported). Moreover, COVID-19 knowledge was shown to predict perceived severity, perceived benefit, and perceived barriers (H1, H3, and H4 were supported). The direct path from knowledge to adherence to preventive behaviors was estimated to be insignificant (H9 was not supported). Overall, these results support the predictions of the health empowerment model, which links knowledge to preventive behaviors through positive attitudes toward the disease.

Several results warrant further mention. First, the effect of knowledge on perceived susceptibility was not statistically significant. This result is consistent with previous studies demonstrating that awareness of a disease risk does not necessarily lead to an increased sense of susceptibility. Fulford, Bunting, Tsibulsky, and Boivin (2013) suggested that knowledge and perceived susceptibility collectively influence one's intention to engage in positive health behaviors. Dillard, McCaul, and Klein (2006) emphasized that most smokers underestimate their vulnerability to smoking, despite having extensive knowledge of how smoking increases the risk of diseases. According to these findings, perceived susceptibility is directly related to health behavior above and beyond the effect of other attitudinal factors, such as perceived severity, perceived benefit, and perceived barriers. Therefore, knowledge and perceived susceptibility should be considered to have an independent impact on behavioral outcomes in the health empowerment model.

This study found that the direct impact of knowledge on preventive behavior was not statistically significant. This result is in line with prior research highlighting the sequential relationship between knowledge, attitude, and behavior. Schrader and Lawless (2004) proposed a model that links knowledge to behavior through attitudes toward issues, arguing that knowledge shapes attitudes about a topic, which in turn affects behavior. Launiala (2009) emphasized that these three factors interact to influence behavior, rather than being a one-way flow. Rav-Marathe, Wan, and Marathe (2016) demonstrated that knowledge and attitude are correlated and jointly determine engagement in preventive practices. However, most existing evidence is cross-sectional and therefore cannot establish any causal inferences. Future research could contribute to the literature by demonstrating the causality and direction of the association between knowledge, attitude, and behavior.

This study could not find significant differences in the association between knowledge, attitude, and behavior based on COVID-19 infection history. Although some paths (i.e., H2 and H8) were marginally different at the p-value of 0.1, the differences were not significant enough to warrant reformulating the health empowerment model with infection history. One possible explanation is that individuals may not have changed their preventive health behaviors in the presence of the risk that they might be re-infected with COVID-19 and suffer from related symptoms (Ghorbani et al., 2022). Previous research on health education has shown that regular education on disease can improve patients' knowledge and attitudes but may not necessarily lead to behavior changes (Clarke, 2009; Vimalavathini, Agarwal, & Gitanjali, 2008). This issue needs to be further tested using a longitudinal study design that tracks changes in preventive health behaviors before and after COVID-19 infection.

One of the key implications of the health empowerment model for health practitioners is the need to assess an individual's knowledge and attitudes towards COVID-19. Practitioners should take into account an individual's background knowledge of COVID-19, as well as their perception of the severity of the symptoms. By understanding an individual's knowledge and attitudes, practitioners can tailor their interventions to promote COVID-19 preventive behaviors and reduce the chance of reinfection.

Another important implication for health practitioners is the need to identify and address perceived barriers to engaging in COVID-19 preventive measures. For example, an individual may be aware of the benefits of health monitoring but may feel that they do not have the willpower to do so. In this case, a health practitioner may need to work with the individual to identify strategies for overcoming these psychological barriers, such as nudge-based mechanism to improve compliance (Nwafor et al., 2021).

Finally, health practitioners need to consider the social and cultural context in which their patients live. The KAB model assumes that attitudes and behaviors are influenced by a range of social and cultural factors, such as family, peers, and media (Mishra & Maity, 2021). Therefore, health practitioners should be aware of these factors and work to address any cultural or social barriers that may prevent their patients from engaging in COVID-19 preventive measures.

This study has several limitations. First, due to the ongoing pandemic in South Korea, data were collected via an online survey. Our sampling procedure does not ensure random sampling of subjects, and therefore our findings may not accurately represent the Korean adult population. Relatedly, our study sample primarily consists of college graduates and married individuals. As a result, our findings may lean towards more educated and married individuals who hold positive attitudes towards infectious diseases and preventive measures. Third, the cross-sectional design of this study makes it infeasible to examine how temporal changes in knowledge and attitudes affect preventive behaviors concerning COVID-19. A longitudinal study exploring the association between knowledge, attitudes, and engagement in preventive behaviors would contribute to understanding how psychological factors influence the chance of COVID-19 infection. Lastly, adherence to COVID-19 preventive measures could have been misreported. Social norms encourage compliance with public health guidelines, so participants were likely to overreport their engagement in COVID-19 preventive behaviors and respond as though they conformed to prevailing social norms (Young & Goldstein, 2021). This misreporting may have introduced attenuation bias in the parameter estimates, resulting in underestimated associations between knowledge, attitudes, and preventive behaviors.

Despite these limitations, this study makes a valuable addition to the literature by demonstrating the potential predictors of COVID-19 preventive behaviors among Korean adults. Our conceptual model that expands the KAB model with the HBM proves its efficacy in predicting individual engagement in preventive practices and uncovering the complex relationships between knowledge, attitudes, and health behaviors. The proposed conceptual framework may be applicable to other infectious diseases where knowledge plays an important role in shaping attitudes and willingness to adopt precautionary measures. Furthermore, this study is one of the first investigations to examine adherence to preventive practices during the transition towards the late phase of the COVID-19 pandemic. Our findings may assist policymakers in establishing public health guidelines for an endemic approach to COVID-19.

### 5.1 Conclusion

This study proposed the expanded health empowerment model to examine public adherence to COVID-19 prevention practices in the late pandemic stage. COVID-19 knowledge was shown to correlate with perceived severity, perceived benefit, and perceived barriers, which are in turn linked to COVID-19 prevention practices. Prior COVID-19 infection did not alter the relationships between knowledge, attitudes, and preventive behaviors. Complying with public health guidelines is the most effective way to contain viruses and reduce mortality. Moreover, understanding the psychological characteristics of individuals who comply with guidelines can inform the development of policies and guidelines aimed at encouraging continued uptake of COVID-19 prevention practices. These findings may assist policymakers and practitioners in designing intervention programs to enhance behavioral adherence to COVID-19 precautionary protocols and provide the necessary infrastructure in the endemic era.

## Ethics approval statement

This study obtained ethical approval from the Jeonju University Research Ethics Committee (JJIRB-220526-HR-20220501). Participants provided informed consent before participating in the study. The data were anonymized and linked by the market research company prior to granting access for research purposes.

## Author statement

Su-Jung Nam: Conceptualization, Data curation, Formal analysis, Funding acquisition, Investigation, Methodology, Project administration, Software, Supervision, Writing - original draft. Tae-Young Pak: Conceptualization, Investigation, Methodology, Project administration, Supervision, Writing - review & editing.

## Data Availability

The data that has been used is confidential.
